# Measles outbreak in complex emergency: estimating vaccine effectiveness and evaluation of the vaccination campaign in Borno State, Nigeria, 2019

**DOI:** 10.1186/s12889-021-10436-1

**Published:** 2021-03-04

**Authors:** Anne Eudes Jean Baptiste, John Wagai, Richard Luce, Balcha Masresha, Don Klinkenberg, Irene Veldhuijzen, Joseph Oteri, Boubacar Dieng, Obianuju Caroline Ikeonu, Sule Meleh, Audu Musa, Fiona Braka, Susan Hahné, E. A. M. Sanders, Eelko Hak

**Affiliations:** 1 World Health Organization (WHO), Country Office, Abuja, Nigeria; 2 World Health Organization (WHO), Inter-country Support Team for West Africa, Ouagadougou, Burkina Faso; 3 World Health Organization (WHO), African Regional Office, Brazzaville, Congo; 4 National Institute for Public Health and The Environment, Bilthoven, Netherlands; 5 National Primary Health Care Development Agency, Abuja, Nigeria; 6 Technical Assistance Consultant, Global Alliance for Vaccines and Immunizations, Abuja, Nigeria; 7 State Primary Health Care Development Agency, Maiduguri, Borno State Nigeria; 8 Groningen Research Institute of Pharmacy, University of Groningen, Groningen, The Netherlands

**Keywords:** Measles, Coverage, Effectiveness, Emergency, Nigeria

## Abstract

**Background:**

From January to May 2019, large measles outbreaks affected Nigeria. Borno state was the most affected, recording 15,237 suspected cases with the state capital of Maiduguri having 1125 cases investigated and line-listed by March 2019. In Borno state, 22 of the 27 Local Government Areas (LGAs or Districts), including 37 internally displaced persons (IDPs) camps were affected**.** In response to the situation, an outbreak response immunization (ORI) campaign was conducted in the 13 most affected LGAs. In addition to conventional vaccination teams, special teams were deployed in security compromised areas, areas with migrants, and for nomadic and IDPs. Here we describe the outbreak and the ORI campaign. We also assess the measles-containing vaccine (MCV) coverage and vaccine effectiveness (VE) in order to quantify the population-level impact.

**Methods:**

We reviewed the ORI activities, and conducted an analysis of the surveillance and the outbreak investigation reports. We assessed VE of MCV by applying the screening-method. Sensitivity analyses were also conducted to assess the effect of final classification of cases on the VE of MCV. The MCV coverage was assessed by a post-campaign coverage survey after completion of the ORI through a quantitative survey in the 12 LGAs that were accessible.

**Results:**

Of the total 15,237 reported measles cases, 2002 cases were line-listed and investigated, and 737 were confirmed for measles by week 9 of 2019. Of the investigated cases 67.3% (*n* = 1348) were between 9 and 59 months of age. Among the 737 confirmed cases, only 9% (*n* = 64) stated being vaccinated with at least 1 dose of MCV. The overall VE for MCV was 98.4% (95%CI: 97.8–98.8). No significant differences were observed in the VE estimates of lab-confirmed and epi-linked cases when compared to the original estimates. The aggregated weighted vaccination coverage was 85.7% (95% CI: 79.6–90.1).

**Conclusion:**

The experience in Borno demonstrates that adequate VE can be obtained in conflict-affected areas. In complex emergencies affected by measles outbreaks, health authorities may consider integration with other health strategies and the engagement of security personnel as part of the ORI activities.

## Background

Between 2017 and early 2019, a global resurgence in measles cases and outbreaks has been observed [1, 2]. Four of the six World Health Organization (WHO) geographic regions reported significant increases in the number and size of measles outbreaks. The most affected countries were Madagascar, Ukraine, Philippines and Nigeria. Reported outbreaks worldwide have been attributed to areas of persistently low measles vaccination coverage [1, 2].

Nigeria has experienced repeated outbreaks of measles in recent years due to low routine immunization (RI) coverage. The country introduced measles vaccination into the RI program in 1978 for children aged 9 months. As at 2005, the national level administrative coverage was 32% [3]. Data from the 2018 Demographic and Health Survey (DHS) indicated that only 54% of children 12–23 months had received measles vaccine [4]; far below the herd protection threshold [5]. From January to May 2019, large measles outbreaks affected all 36 states in Nigeria and the Federal Capital Territory (FCT). By the beginning of May 2019, there were more than 28,000 suspected measles cases reported and 89 associated deaths with measles. A majority of cases were reported in Borno State with over 15,000 suspected cases and 75 measles deaths across 37 internally displaced persons (IDP) camps in 22 Local Government Areas (LGAs or Districts). High measles-associated mortality rates have been previously reported from IDP camps, and measles has been one of the major causes of child deaths in humanitarian emergencies [6, 7].

To respond to measles outbreaks in emergencies, the WHO recommends a range of epidemic control activities. These include review of epidemiological and immunization program data to identify the cause (s) of the outbreak, intensive measles surveillance, improving coverage amongst high risk populations and implementing supplementary immunization activities (SIA) in areas not yet affected by the outbreak [8]. In addition, complex humanitarian emergencies pose considerable logistical challenges to the delivery of vaccination services including maintaining appropriate cold chain conditions for vaccine storage. High vaccine effectiveness (VE) is critical to halt outbreaks and prevent ongoing transmission during complex emergencies. Thus, estimating the VE of measles-containing vaccine (MCV) following a large measles outbreak is an essential part of assessing the quality and efficacy of the response [9, 10].

During the 2019 measles outbreak in Borno state, the government of Nigeria through its National Primary Health Care Development Agency (NPHCDA), conducted two phases (March and May) of measles outbreak response immunization (ORI) in the most affected areas of the state. Here we describe the outbreak response and its impact in Borno State by assessing the investigated and confirmed measles cases as well as their vaccination status. To assess the population-level impact, we estimated the age-specific VE for MCV by applying the screening method on these data, and by estimating the vaccination coverage using a post-campaign coverage survey (PCCS).

## Methods

### Setting

Borno State is located in North East of Nigeria with a land mass of 75,481 km^2^ and a population of about 6.4 million people (2019 population census projections) [11]. The state shares an international boundary with Niger Republic, Chad and Cameroon. It shares interstate borders with Yobe (to the West), Adamawa (to the South), and Gombe (to the South West). Borno has 27 LGAs comprising a total of 311 political wards, out of which 201 are accessible for immunization and surveillance activities, and were fully accessible at the time of the ORI. The 2018 DHS indicated that 46.5% of children aged 12–23 months were vaccinated with MCV in Borno [4].

Since 2009, an armed insurgency has been ongoing in Borno State. This has resulted in the displacement of communities and the disruption of health services due to destruction of health facilities and out-migration of health personnel. The last weeks of December 2018 witnessed increased attacks on civilian populations by armed groups and resulted in a massive population displacement from security-compromised areas, including inaccessible settlements (communities), into the metropolitan LGAs of the state. By January 2019, more than 700,000 people were living in overcrowded camp-like settings, significantly increasing the risk of epidemics and infectious diseases. As a result, approximately 20% of the population of Borno State are living in IDPs camps in hosted communities [12].

### Case reporting and definition of outbreak and cases

In Nigeria, measles case-based surveillance is integrated with the polio acute flaccid paralysis surveillance structure and includes four national measles serological laboratories capable of testing on specimens from suspected measles cases for immunoglobulin (Ig) M antibodies as described elsewhere [13]. The surveillance performance is monitored regularly using performance monitoring indicators (i.e. proportion of LGAs with at least 1 suspected measles case reported with a blood specimen in a year, proportion of reported suspected cases from whom blood specimen is collected, non-measles febrile rash illness rate, and incidence of confirmed measles per million population). The measles surveillance system is sufficiently sensitive to identify and confirm measles cases. With one exception (i.e. incidence of confirmed measles per million population), the annual targets for the core measles surveillance performance indicators have been met since 2017 [14]. In brief, the surveillance network runs from the settlements and wards through the LGAs and States to the National level. The network consists of disease surveillance and notification officers (DSNOs) in all the LGAs in the country with State Epidemiologists at the State level. It is therefore institution-based and consists of formal and informal healthcare delivery points (reporting sites) and non-formal health care providers (informants). Additional to the installed surveillance network, a community-based surveillance makes use of community informants (i.e. traditional birth attendants, patent drug vendors, traditional healers) to report suspected cases.

A measles outbreak was defined as the occurrence of three or more confirmed measles cases in a unit (i.e. health facility/LGA/community with an approximate catchment population of ≥100,000) in a month [8, 15]. We reviewed measles outbreak investigation reports and measles cases line-listed by the DSNO, WHO Borno State office and the rapid response team (RRT) deployed to Borno. Data collected included name, address (i.e. rural vs urban, LGA, ward, settlement/community), age, sex, date of rash onset, vaccination status, date of last vaccination, date of specimen collection and, final classification of cases as at epidemiologic week 9. Additional data was collected from the measles case based surveillance database regarding date of rash onset, age, vaccination status, lab-confirmed – positive serologic test for measles IgM antibody, epidemiologic linkage, and clinically compatible cases. WHO case definitions for measles were used for final classification of cases [16].

### Measles outbreak response immunization – ORI

To improve population immunity and interrupt the large measles outbreak, the ORI was conducted in 2 phases. An objective of the ORI was to reach the underserved children aged between 6 months and 71 months. Key elements considered by NPHCDA to determine the target population and scope of the vaccination activities included routine vaccination coverage, age-specific of lab-confirmed cases, absolute number of cases (suspected and lab-confirmed) and, results of the measles risk assessment. Phase 1 targeted children in 8 wards of the state capital of Maiduguri Metropolitan Council (MMC) from the 21st to the 25th of March 2019.

Phase 2 was implemented from the 14th to the 21st of May 2019, and included accessible wards of 13 LGAs out of the 27 LGAs of Borno States. In addition to conventional vaccination teams, special teams were used during the response. These teams, with the support of local vigilante and military, were deployed during the 2nd phase of the campaign to safely operate in security compromised areas, areas with migrant and other special populations (i.e. nomadic, IDPs in camps and those embedded host community). The processes of engagement of security personnel to access security-compromised areas was described elsewhere [17]. A total of 37 special teams were used to reach underserved children in partially accessible and/or security compromised areas of the LGA.

### Estimation of measles vaccine effectiveness (VE)

Measles VE was calculated using the screening method according to the following formula: VE = 1-((PCV/(1-PCV)) * (1-PPV)/PPV); where PCV is the proportion of cases vaccinated and PPV is the proportion of the population vaccinated. The screening method estimates VE by comparing vaccination coverage in cases of a disease (PCV) with the vaccination coverage in the population from which the cases are derived (PPV). PCV was calculated from the outbreak investigation reports and the measles surveillance database, and PPV was computed from the 2018 PCCS report. Evaluation of VE was assessed in children 9 months to 71 months of age for a more accurate estimate of the VE. Infants below the age of 9 months were not scheduled for measles vaccination and were excluded from the VE estimation. Likewise, cases reported from IDP camps were excluded from the VE estimation as the PCCS did not capture coverage from IDP camps.

### Sensitivity analysis

We conducted a sensitivity analysis where we compared the VE for lab-confirmed cases with the VE for epi-linked cases. As the original VE calculation, we did not considered cases reported from IDP camps nor vaccination of under 9 months. The sensitivity analysis was performed to assess the effect of final classification of cases on the MCV VE estimate.

### PCCS – sample design

We designed a quantitative survey assessment of the ORI after completion of the 2nd phase of the ORI. The survey was a two-stage cluster household survey. A unique sample design was applied in the Borno PCCS. Due to absence of a recent sampling frame and limited funds to conduct household listing, the aggregate of ward micro-plans, which contained the number of settlements in a ward and the estimated population per settlement, was used as the sampling frame for this survey. The settlement was randomly selected as the primary sampling unit and individual households as secondary sampling units. The primary sampling frame consisted of 1044 settlements from which 120 primary sampling units (settlements) were selected in the 12 accessible LGAs at the time of the survey. The secondary sampling involved the selection of households within each settlement by using systematic random sampling. All the households in a settlement were visited, and eligible households with children aged 9 months to 71 months identified and line listed formed the settlement sampling frame. We calculated the sampling interval by dividing the total number of eligible households in the settlement with the desired cluster household size (15). Subsequently, we randomly selected a starting point on the line list and all subsequent households after addition of the sampling interval. Therefore, a sample of 15 households was randomly selected in each settlement providing a total of 1800 households in 12 out of the 13 LGAs involved in the ORI (one LGA [Biu] was not surveyed due to insecurity).

### PCCS – data collection

We collected data using Open Data Kit software running on android mobile phones. On completion of the household roster, only age-eligible respondents were present for interviewing. Information on the children receiving measles vaccination (i.e. children aged 9 to 71 months with SIA cards and recall) including vaccination coverage during the measles campaign were collected. Design weights were calculated as the product of inverse probabilities of selection in the first and second stages. Data collection was conducted between 7th and 19th of July 2019.

### PCCS – data cleaning and analysis

We used the SIA module of Vaccination Coverage Quality Indicators software running on Stata version 15 (StataCorp. 2017) for data cleaning and analysis. Stata Statistical Software: Release 15. College Station, TX: StataCorp LLC) [18]. Results are based on the weighted data to account for the survey sampling design and non-response. Wilson’s 95% confidence intervals and upper and lower confidence bounds have been computed.

## Results

### Description of the outbreak and the response

**Fig. 1:**
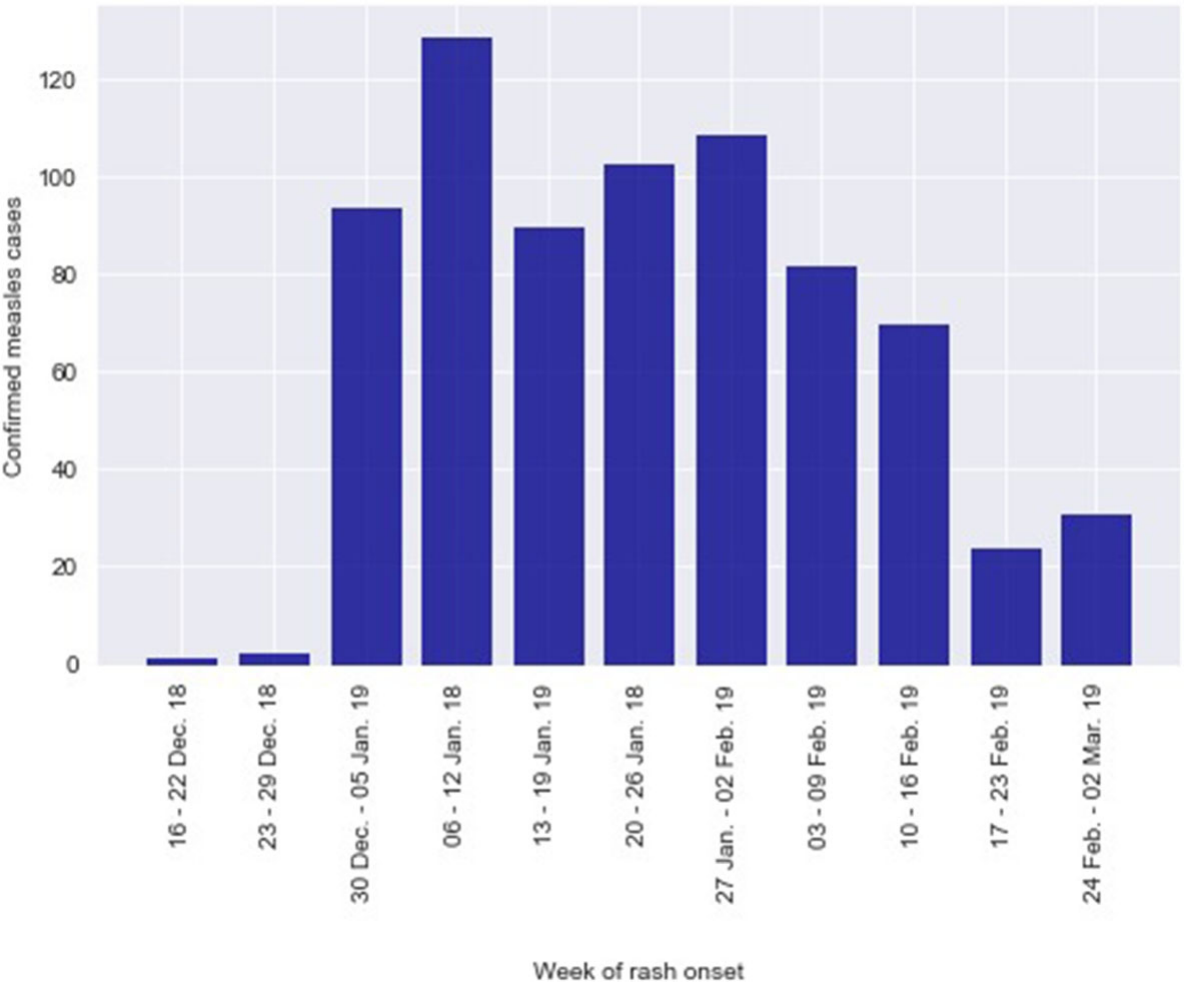
Confirmed cases of measles by week of rash onset, Borno State, Nigeria, December 2018 – March, 2019. As of epidemiologic week 9, a total of 2002 cases were investigated and line-listed. Among the investigated cases, 737 were confirmed for measles

The number of reported cases of measles per week of onset began to increase in week 2 and remained elevated until week 9, 2019. Of the total 15,237 reported cases from Borno, 2002 suspected cases were line-listed and investigated from 17 LGAs as of March 2019 (epidemiologic week 9), and 737 were confirmed (103 IgM positive by laboratory testing and 634 epi-linked) (Fig. 1 and Table 1). Of the investigated cases, 1348 (67.3%) were between 9 and 59 months of age; 145 (7.2%) were infants aged 9–11 months.

**Table 1:** Number and proportion (%) of investigated reported cases by age-group and vaccination status. Borno, December 2018 – March, 2019

Age group	Cases reported		Confirmed cases*							
			All		Unvaccinated		Unknown vaccination		Vaccinated	
	No.	%	No.	%	No.	%	No.	%	No.	%
< 9 months	169	8.4%	61	8.3%	61	9.3%	0	0.0%	0	0.0%
9–11 months	145	7.2%	49	6.6%	38	5.8%	3	15.0%	8	12.5%
12–23 months	429	21.4%	148	20.1%	134	20.5%	3	15.0%	11	17.2%
24–35 months	355	17.7%	127	17.2%	106	16.2%	2	10.0%	19	29.7%
36–47 months	251	12.5%	98	13.3%	87	13.3%	5	25.0%	6	9.4%
48–59 months	168	8.4%	65	8.8%	51	7.8%	3	15.0%	11	17.2%
60–71 months	124	6.2%	54	7.3%	50	7.7%	0	0.0%	4	6.3%
> 71 months	361	18.0%	135	18.3%	126	19.3%	4	20.0%	5	7.8%
**Total**	**2002**	**100%**	**737**	**100%**	**653**	**100%**	**20**	**100%**	**64**	**100%**

Source: Outbreak investigation report and Measles Line-list

^*^Confirmed cases = Lab-confirmed + EpiLink + Clinical compatible

**Fig. 2:**
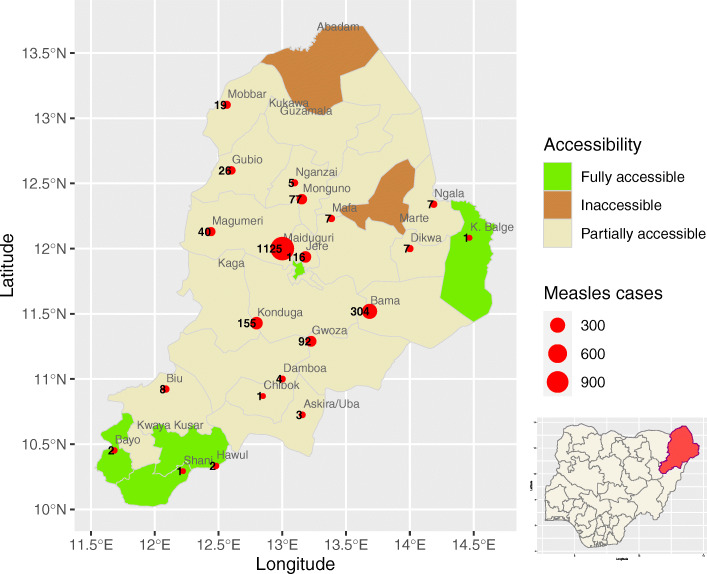
Reported measles cases. Borno state, Nigeria, December 2018 – March, 2019. Measles cases = Cases investigated and line-listed; Source: Borno State measles line-list. The number of measles cases were greater in Maiduguri (attack rate per 100,000: 50.70), followed by Bama (attack rate per 100,000: 331.43), Konduga (attack rate per 100,000: 28.14) and Jere (attack rate per 100,000: 14.19). Challenges around accessibility to various communities to implement and provide health services remain a barrier in Borno State. At the time of the outbreak response immunization, two LGAs (i.e. Abadam and Marte) were totally inaccessible and, only four LGAs (out of the 27) and the state capital (Maiduguri) were fully accessible. The remaining LGAs were partially accessible. We plotted the maps using R software and shape files obtained from open source website. http://www.gadm.org. The shape files have not been previously copyrighted to our knowledge. Date created: December 2020

Among the 737 confirmed cases (all age groups), 64 cases (9%) stated (by recall and vaccination card) being vaccinated with at least 1 dose of measles-containing vaccine. Eight cases from 9 to 11 months were vaccinated for measles (Table 1). The proportion of unvaccinated cases was higher among the 12–23 months (134 cases) and 24–35 months (106 cases). The geographic distribution of investigated/line listed cases are shown in Fig. 2. The number of reported cases were greater in MMC (attack rate per 100,000: 50.70), followed by Bama (attack rate per 100,000: 331.43), Konduga (attack rate per 100,000: 28.14) and Jere (attack rate per 100,000: 14.19).

The first phase of the ORI in MMC was conducted in epidemiologic week 11 and during week 20 in the remaining LGAs. The number of children vaccinated with measles vaccine was 437,515 (administrative coverage: 94%) during phase 1, and 800,666 (administrative coverage 95%) children during phase 2.

### Vaccine effectiveness

A total of 468 cases were confirmed from the age group 9 to 71 months and among them, 47 were vaccinated. At 9–11 months of age, 12–23 months and 24–35 months, the VE was 87.3% (95%CI: 71.0–95.2), 96.2% (95%CI: 92.6% – 98.3) and 97.3% (95%CI: 95.1–98.7) respectively (Table 2). The highest VE was nearly 100% for the age group 60–71 months (99.5%; 95%CI: 98.3–99.9). The overall VE for MCV was 98.4 (95%CI: 97.8–98.8).

**Table 2:** Age-specific vaccine effectiveness of confirmed measles cases. Borno, December 2018 – March, 2019

Age Group	Vaccinated cases	Confirmed^a^ cases	PCV (95% CI)	PPV	VE % (95% CI)
< 9 months^b^	1	49	0.02 (0.00–0.11)	0.00	–
9–11 months	7	42	0.17 (0.07–0.31)	0.61	87.3 (71.0–95.2)
12–23 months	9	118	0.08 (0.04–0.14)	0.69	96.2 (92.6–98.3)
24–35 months	12	97	0.12 (0.07–0.21)	0.84	97.3 (95.1–98.7)
36–47 months	5	75	0.07 (0.02–0.15)	0.84	98.6 (96.7–99.6)
48–59 months	10	49	0.20 (0.10–0.34)	0.70	89.2 (77.9–95.2)
60–71 months	3	38	0.08 (0.02–0.21)	0.94	99.5 (98.3–99.9)
**Overall**	**47**	**468**	**0.10 (0.07–0.13)**	**0.88**	**98.4 (97.8–98.8)**

*PCV* Proportion of cases vaccinated, *PPV* Proportion of the population vaccinated based on 2018 PCCS report, *VE* Vaccine effectiveness, *CI* Confidence Interval.

^a^Cases reported from IDP camps were excluded from the VE estimation as the PCCS did not capture coverage from IDP camps

^b^The < 9 month old infants were not scheduled for measles vaccination and were excluded from the VE estimation

### Sensitivity analysis

When estimating the VE by final classification of cases, 44 epi-linked cases were accounted as receiving one or two doses of MCV and 3 lab-confirmed cases were found receiving one dose. The VE was then estimated at 68.3% (95%CI: − 509 – 99.5) for lab-confirmed cases and 88.5% (95%CI: 72.1–96.1) for epi-linked cases of children 9 to 11 months. The VE for lab-confirmed cases was low as the PCV (only 1 vaccinated cases out of the 3 lab-confirmed cases; PCV: 0.33, 95%CI: 0.01–0.91) was high.

When at 12 to 23 months, the VE estimate was 95.9% (95%CI: 71.5–99.9) for lab-confirmed cases and 96.3% (95%CI: 92.4–98.4) for epi-linked cases; The original VE was estimated at 96.2% (95%CI: 92.6–98.3) for this age group. No significant differences were observed in the VE estimates of the two scenarios (lab-confirmed vs epi-linked) when compared to the original estimates; with the exception of the 9 months to 11 months for lab-confirmed (Table 3).

**Table 3:** Sensitivity analyses of lab-confirmed and epi-linked cases, and age-specific vaccine effectiveness. Borno, December 2018 – March, 2019

Original VE		Sensitivity analyses							
		VE for lab-confirmed cases				VE for epi-linked cases			
**Age Group**	**VE% (95% CI)**	**Vaccinated cases**	**Confirmed**^a^ **cases**	**PCV (95% CI)**	**VE% (95% CI)**	**Vaccinated cases**	**Confirmed**^a^ **cases**	**PCV (95% CI)**	**VE% (95% CI)**
< 9 mo^b^	–	0	2	0.00 (0.00–0.84)	–	1	47	0.02 (0.00–0.11)	–
9–11 mo	87.3 (71.0–95.2)	1	3	0.33 (0.01–0.91)	68.3 (−509–99.5)	6	39	0.15 (0.06–0.31)	88.5 (72.1–96.1)
12–23 mo	96.2 (92.6–98.3)	1	12	0.08 (0.00–0.38)	95.9 (71.5–99.9)	8	106	0.08 (0.03–0.14)	96.3 (92.4–98.4)
24–35 mo	97.3 (95.1–98.7)	0	11	0.00 (0.00–0.28)	100.0 (92.4–100.0)	12	86	0.14 (0.07–0.23)	96.9 (94.3–98.5)
36–47 mo	98.6 (96.7–99.6)	0	7	0.00 (0.00–0.41)	100.0 (86.8–100.0)	5	68	0.07 (0.02–0.16)	98.5 (96.3–99.5)
48–59 mo	89.2 (77.9–95.2)	1	8	0.13 (0.00–0.51)	94.0 (53.0–99.9)	9	41	0.22 (0.11–0.38)	88.1 (74.5–95.0)
60–71 mo	99.5 (98.3–99.9)	0	7	0.00 (0.00–0.41)	100.0 (95.6–100.0)	3	31	0.10 (0.02–0.26)	99.3 (97.8–99.9)
**Overall**	**98.4 (97.8–98.8)**	**3**	**50**	**0.06 (0.01–0.17)**	**99.1 (97.2–99.8)**	**44**	**418**	**0.11 (0.08–0.14)**	**98.3** **(97.7–98.8)**

*Mo* month, *PCV* Proportion of cases vaccinated, *PP* Proportion of the population vaccinated based on 2018 PCCS report, *VE* Vaccine effectiveness, *CI* Confidence Interval

^a^Cases reported from IDP camps were excluded from the VE estimation as the PCCS did not capture coverage from IDP camps

^b^The < 9 months’ infants were not scheduled for measles vaccination and were excluded from the VE estimation

### Post campaign coverage survey

The proportion of individuals who received measles vaccination during the ORI by source of information on vaccination status and age group are shown in Table 4. The aggregated weighted coverage is 85.7% (95%CI: 79.6–90.1). Children under 9 months and 24 to 35 months age group had the highest coverage at 100 and 92.8% respectively. While 80% of all respondents reported having received vaccination cards during the campaign, only 32.2% (95%CI: 25.0–40.3) had card evidence for their vaccination status. Fifty-three percent (53.5%) of individuals who were vaccinated had no card evidence for their vaccination.

**Table 4:** Post measles campaign survey by card and by recall. Borno^a^, July 2019

	Vaccinated by recall		Vaccinated by card^b^		Vaccinated by card or recall		N	Weight
	%	CI	%	CI	%	CI		
**Borno**	**53.5**	**(45.4–61.4)**	**32.2**	**(25.0–40.3)**	**85.7**	**(79.6–90.1)**	**1440**	**1440**
**Age category**								
< 9 months	28.8	(4.3–78.4)	71.2	(21.6–95.7)	100.0	(20.7–100)	6	2
9–11 months	52.6	(25.2–78.6)	33.5	(13.4–62.2)	86.2	(61.9–96.0)	31	15
12–23 months	50.3	(36.0–64.5)	28.6	(18.7–41.1)	78.9	(58.5–90.9)	283	308
24–35 months	54.9	(43.8–65.6)	37.8	(27.5–49.5)	92.8	(87.2–96.0)	368	333
36–47 months	43.4	(33.0–54.4)	42.9	(31.7–54.8)	86.2	(77.6–91.9)	376	387
48–59 months	61.8	(50.8–71.7)	22.4	(14.6–32.7)	84.2	(73.8–91.0)	308	296
60–71 months	66.7	(28.9–90.8)	11.5	(3.2–34.2)	78.2	(30.1–96.8)	42	28
> 71 months	75.7	(37.4–94.2)	11.7	(1.9–46.8)	87.3	(56.7–97.3)	26	71

*CI* Confidence Interval, *N* Number interviewed

^a^Thirteen (13) LGAs out of the 27 LGAs of Borno States were involved in the ORI. Fifteen (15) of the 27 LGAs of Borno State were not included in the survey

^b^80% of all respondents reported having received vaccination cards during the campaign

## Discussion

Increased attacks in Borno State in December 2018 led to a massive population displacement. Subsequently, there were an increasing number of unvaccinated children who moved toward MMC and living in IDP camps in conditions favoring measles transmission. An ORI was essential for improving population immunity and interrupting the large measles outbreak in line with the global measles and rubella control and elimination goals [19].

The State has a history of outbreaks of measles in the last 5 years due to persistently low routine immunization coverage, and has been conducting measles campaigns almost every 2 years as a key strategy for reaching high-risk populations in security challenged/hard-to-reach areas. In November 2015, Borno responded to a measles outbreak targeting 1,255,100 children aged 6 to 59 months for vaccination with MCV. Only 66.7% (837,743 children) of the targeted children were vaccinated during the response. In early 2017, a mass measles vaccination campaign was implemented as part of outbreak preparedness in conflict-affected areas of Borno state. The campaign was implemented in 25 LGAs of the State. According to the PCCS concluded in February 2018, the proportion of children aged 9 months to 59 months who received measles vaccine during the 2017 campaign was 72.2% and, of all respondents, only 52.2% had received measles vaccines before the campaign [20].

During the 2019 ORI, a total of 1,238,181 children were vaccinated with MCV giving an administrative coverage of 98.75%. As administrative coverage reports may not always be accurate, a PCCS was conducted and has indicated an aggregated weighted coverage for the 12 LGAs included in the survey of 85.7%. The 95% coverage objective was not achieved due to the complexity in conducting a vaccination campaign in an emergency setting where insecurity, regular influx of new persons, population displacement, and hard-to-reach areas are extensive. In order to overcome these obstacles, collaboration with the military enabled the vaccination teams to immunize eligible children in security compromised areas. This strategy builds upon the Reaching Inaccessible Children strategy (RIC) conducted by the military and the Reaching Every Settlement (RES) strategy which uses some military support to reach areas that were partially or completely inaccessible to the vaccination teams [17]. In recently accessed territories at the time of the ORI (i.e. camps and communities in Bama, Gwoza, Dikwa, Damboa, Ngala, Kukawa and Monguno LGAs), “hit and run” vaccination activities have been conducted with military escort to provide immunization and basic health care in the camps in these areas. Additionally, in hard-to-reach settlements, the polio eradication platform was used to reach underserved communities with measles vaccination [21].

Data on measles VE estimations in emergency settings in Nigeria and other countries are limited, and, where available, quite dated. During a measles outbreak in refugee camps in Mozambican refugee camps in Malawi, VE was estimated for children less than 5 years using the screening method. The findings of this investigation showed a VE of more than 90% [22]. A two-stage cluster survey of 563 children in famine emergencies in Ethiopia found a low VE of 66.9% in children 9–36 months old. The authors suggested problems with the cold chain or vaccine administration [7]. We have obtained a high estimate of MCV VE among all age groups during ORI in Borno state Nigeria. VE ranged from 87.3% (95%CI: 71.0, 95.2) to 99.5% (95%CI: 98.3–99.9) for children from 9 to 71 months. Measles VE at 9–11 months and in those greater than 12 months of age is expected to range between 84 and 92.5% [5].

Despite the limited storage capacity and poor immunization infrastructures at the lower level, efforts were made to ensure adequate cold chain and vaccine handling. Upon receipt of the MCV in-country, the vaccines were prepositioned in the Zonal cold store in Bauchi State (452 km driving distance to MMC). The vaccines and devices were distributed from the Zonal to the State store 2 weeks before the commencement of the ORI. The State distributed bundled vaccines to the LGA stores ahead of the campaign using cold boxes. Fast-cold chain was used to comply with the vaccine distribution process up to the communities and service delivery points.

This study is subject to some limitations. First, the result of the PCCS survey cannot be generalized to all children in the eligible age group in Borno state. The PCCS coverage results do not include coverage for children in the 14 LGAs that were not included in the survey since they were not part of the ORI and the additional LGA (Biu) dropped from the survey due to insecurity. The survey teams did not visit IDP camps and in addition, as a consequence of the ongoing insecurity, there has been constant displacement and migration of populations within and out of Borno state. The number of children eligible for vaccination at any settlement in Borno state is constantly changing and it is difficult to track movements and how the movements may affect the proportion of vaccinated and unvaccinated children. Limiting the analysis to non-IDP cases may under-estimate the PPV of the case population. Secondly, the vaccination status of children in the inaccessible wards could not be estimated. The final limitation of the PCCS is (due to security challenges) the lag time of 4 months and 2 months between completion of the two phases of the campaign and timing of the survey. This may have influenced survey results as a consequence of recall bias. Only 32% of all interviewed individuals produced their vaccination cards, and 20% did not receive cards or could not recall whether they received vaccination cards. Considering multiple antigens delivered in the RI schedule and periodic ORI and preventive supplementary immunization activities, it is difficult for parents to accurately recall all doses of all antigens received by their child in the absence of widespread retention of vaccination cards. However, as demonstrated by previous immunization surveys conducted in Nigeria, using cards alone actually underestimates coverage, if not all doses are recorded [23].

In addition to the above mentioned limitations, there are some potential biases in all observational VE studies including the screening method [24]. Specifically, bias related with misclassification and definition of cases, and recording of vaccination status. In our study, these biases were minimized by using the WHO standard case definition for measles, and the cases that were investigated and line-listed by the surveillance officer (i.e. DSNO).

## Conclusions

Our findings suggest that decreased VE is unlikely to explain the recurrence of measles outbreaks in Borno State. Insecurity resulting in lack of health and immunization services, frequent population displacements and clustering of susceptible children in IDPs are more likely contributors to measles morbidity and mortality in the State [25]. Despite the estimated VE from the ORI activities, sub-optimal RI coverage is insufficient to maintain population immunity and prevent the accumulation of susceptible children that allow outbreaks to occur.

This analysis provides important insight for future vaccination in emergency and humanitarian settings in Nigeria and elsewhere. Data on the target age range for vaccination campaigns and strategies for measles control in response to an outbreak of measles in complex emergencies are available [26–28]. Adequate VE can be obtained with appropriate cold chain capacity when conducting ORI in situations where insecurity may challenge vaccine conservation. The integration with other health strategies (i.e. polio platform) and the engagement of security personnel increased the number of communities that could be accessed and reduced the time spent in these areas by the vaccination team. As measles has been one of the major causes of child deaths in complex emergencies, regular assessment of local measles epidemiology and population immunity status is recommended to determine the risk of an outbreak [6, 27].

## Data Availability

Some of the data sets used in this paper are publically available via the sources referenced in the manuscript (i.e. Nigeria Demographic and Health Survey 2018). Data such as the patient’s names (from the measles case-based and the outbreak investigation reports) are confidential and the authors do not have the permission to share these data. Data for the post campaign coverage survey are available and can be obtain on request from the WHO Country Office (WCO) in Nigeria.

## Abbreviations

DSNOs: Disease Surveillance and Notification Officers
FCT: Federal Capital Territory
IDPs: Internally Displaced Persons
Ig: Immunoglobulin
LGAs: Local government areas
MCV: Measles-containing vaccine
MMC: Maiduguri Metropolitan Council
NPHCDA: National Primary Health Care Development Agency
PCCS: Post-campaign coverage survey
PCV: Proportion of cases vaccinated
PPV: Proportion of the population vaccinated
RES: Reaching Every Settlement
RIC: Reaching Inaccessible Children strategy
RRT: Rapid Response Team
SIA: Supplemental immunization activities
VE: Vaccine Effectiveness
WHO: World Health Organization
